# Prevention and management of CVD in LMICs: why do ethnicity, culture, and context matter?

**DOI:** 10.1186/s12916-019-1480-9

**Published:** 2020-01-24

**Authors:** Shuchi Anand, Christina Bradshaw, Dorairaj Prabhakaran

**Affiliations:** 1 Stanford University School of Medicine, Palo Alto, CA USA; 2 Center for Chronic Condition and Injuries, Public Health Foundation of India, 6th Floor, Plot No. 47, Sector 44, Gurgaon, 122002 India; 3 Department of Epidemiology, London School of Hygiene and Tropical Medicine, London, UK; 4 Rollins School of Public Health, Emory University, Atlanta, GA USA

**Keywords:** Cardiovascular disorders, Low- and middle-income countries, Race/ethnicity

## Abstract

**Background:**

Low- and middle-income countries now experience the highest prevalence and mortality rates of cardiovascular disease.

**Main text:**

While improving the availability and delivery of proven, effective therapies will no doubt mitigate this burden, we posit that studies evaluating cardiovascular disease risk factors, management strategies and service delivery, in diverse settings and diverse populations, are equally critical to improving outcomes in low- and middle-income countries. Focusing on examples drawn from four cardiovascular diseases — coronary artery disease, stroke, diabetes and kidney disease — we argue that ethnicity, culture and context matter in determining the risk factors for disease as well as the comparative effectiveness of medications and other interventions, particularly diet and lifestyle interventions.

**Conclusion:**

We believe that a host of cohort studies and randomized control trials currently being conducted or planned in low- and middle-income countries, focusing on previously understudied race/ethnic groups, have the potential to increase knowledge about the cause(s) and management of cardiovascular diseases across the world.

## Background

The majority of the rich, decades-long epidemiology data on risk factors for cardiovascular disease (CVD) has been obtained from studies in high-income settings [1–4]. These studies subsequently created the fundamental knowledge base to propel highly effective CVD interventions, including life-style interventions, antihypertensives, lipid-lowering statins, and antiproteinuric renin-angiotensin system blockers. However, a substantial burden of CVD persists, which is much higher in low- and middle-income countries (LMICs) than in high-income countries [5]; close to 80% of CVD deaths occur in LMICs, and nearly 40% of these are labeled as premature [5]. This is despite the lower prevalence of cardiovascular risk factors such as high blood pressure, obesity, diabetes and dyslipidemia in LMICs. One obvious explanation is the lack of resources to effectively deliver proven therapies. Herein, we lay out a case for probing beyond resource paucity as the chief driver for variation in cardiovascular risk and/or outcomes in diverse populations in the world.

**Fig. 1:**
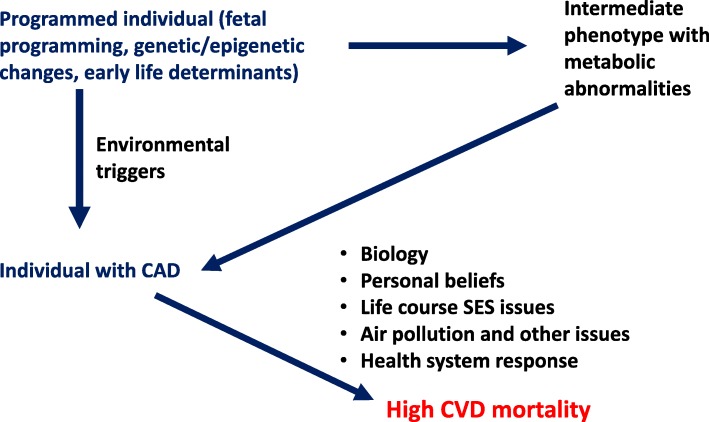
Conceptual basis for ethnicity and cultural context in cardiovascular disease (CVD). *SES* socioeconomic status

Focusing on four categories of CVD — coronary artery disease, stroke, diabetes and kidney disease — we highlight recently established or emerging risk factors for CVD retrieved from studies performed in ethnically diverse populations. We also discuss the importance of studies evaluating comparative effectiveness of established therapies given data demonstrating differences in response by race/ethnicity. Finally, we provide the rationale for clinical trials evaluating region-specific and innovative models of care delivery. All of this work, we believe, has the potential to expand the scientific knowledge base informing strategies to combat CVD. Figure 1 lays out the conceptual basis for our argument.

## Risk factors for CVD: why we need studies in diverse populations, in diverse regions of the world

While clinicians have long recognized that prevalence of specific CVDs varies by race, ethnicity or geography (Table 1 demonstrates, for example, the high and growing prevalence of hypertension in low-income countries), only recently has there been intensive research focus to investigate the reasons for these variations; whether these are genetically or culturally based, understanding them has been ‘game-changing’ for some fields.

**Table 1:** Prevalence of risk factors by country income groups

Risk factors, % of population	Low income	Low middle income	Upper middle income	High income
Tobacco use, men	31	32	43	33
Tobacco use, women	3	3	5	18
Insufficient physical activity	17	17	25	33
Overweight	21	27	43	56
High blood pressure	28	25	20	19
High cholesterol	24	32	46	59
High fasting glucose	7	9	9	7

Populations referred to are adults 18 years or older, except for tobacco use (15 years or older) and high cholesterol (25 years or older). Overweight defined as body mass index ≥25 kg/m^2^, high blood pressure as ≥140 mmHg (systolic) or > 90 mmHg (diastolic), high cholesterol as ≥5.0 mmol/L, and high fasting glucose as ≥7.0 mmol/L.

Adapted from [6]

One of the most prominent examples arises from the kidney disease literature. It was well-established that African Americans have up to fourfold the risk for end-stage kidney disease compared with Caucasians [7], with hypertensive nephrosclerosis [8] and focal segmental glomerulosclerosis [9] occurring at much higher frequencies in African Americans. A decade-long search for a possible genetic basis led to the discovery of Apolipoprotein (APOL)-1 polymorphisms [10, 11], present in up to 30% of some ethnic groups in West Africa [12], which concurrently protect against sleeping sickness but confer a higher risk for hypertensive nephrosclerosis or focal segmental glomerulosclerosis in West African compared with Caucasian or East African populations. This discovery has not only changed our understanding of risk for end-stage kidney disease in African Americans (prior hypotheses had largely centered around access to care), it is also affecting risk stratification for kidney transplant [13]. A difference in the causes of kidney disease but not necessarily overall prevalence exists for other ethnicities and regions [14], including for IgA nephropathy, the most common cause of glomerulonephritis in the world and which has a well-established predilection for Asian and Mediterranean populations. Intense efforts are underway to identify a genetic basis [15].

The prevalence and risk for diabetes also varies widely by ethnicity. In Hispanic and Latino populations in the USA, diabetes prevalence nears twofold that of US Caucasians [16]. Genome-wide analysis studies focused specifically on the Mexican and Mexican American populations identified a candidate locus that could result in disordered lipid metabolism; this locus occurs at high frequency in Native American and East Asian populations but is rare in Caucasians [17]. Other work from the Pima Indian population — nearly 50% of whom have diabetes — identified a locus that predisposes to high birthweight and confers a twofold increase in the risk for type 2 diabetes [18]. A recent study comparing insulin profiles in two high-risk ethnic groups found a higher propensity for insulin resistance, as measured by HOMA-IR, among Pima Indians compared with Asian Indians, who had substantially lower insulin secretion [19]. Thus, understanding the mechanisms (genetic, fetal programming or environmental) that operate in a given ethnic group could help stratify whether patients are likely to have perturbations in insulin secretion versus resistance, and thus allow for tailored initial therapy.

Studies evaluating risk factors in diverse populations can also highlight cross-cultural differences in diet or lifestyle that result in variations in disease patterns. In an early study comparing mortality and risk factors in three regions of the world (Finland, China, and USA) [20], the authors discovered striking differences in total cholesterol (highest in Finland and lowest in China) and ischemic heart disease mortality (age-standardized mortality rate nearly tenfold higher in Finland than in China). On the other hand, dietary salt intake and age-standardized stroke mortality was substantially higher in China in this and in many other larger analyses [21]. The Prospective Urban RurEpidemiology (PURE) study is perhaps one of the largest and longest running examples of an attempt to explore the complex interactions between cultural and genetic CVD risk factors and the likelihood of events in 17 countries across the income spectrum [22]. Despite a lower prevalence of traditionally recognized risk factors in LMICs, PURE investigators observed higher event rates in LMICs than in high-income countries [23], providing impetus to investigate ‘non-traditional’ risk factors that could be contributing to higher event rates in LMICs.

Several hypothesized non-traditional risk factors, such as low birthweight, air pollution, and occupational exposures, including to pesticides and heat stress, may contribute to burden of disease across the world but could be better studied in LMICs due to the higher likelihood of exposure in these settings. The US National Institutes of Health Fogarty Institute has funded seven ‘Geo-health’ collaborations across the world to evaluate the contribution of environmental (e.g., outdoor and indoor air pollution) and occupational exposures (e.g., in street vendors or electronic waste recycling workers) to human disease. International consortia have formed to investigate the cause of a chronic kidney disease of unknown etiology among agricultural workers living in lowland areas [24], a disease more common in certain LMICs but possibly occurring at lower intensity and/or frequency in high-income countries as well.

## Interventions for CVD: why drugs and models of care may need region-specific fine tuning

Similar to epidemiologic data on CVD, the evidence for its therapeutic interventions has largely been derived from high-income settings. However, just as CVD phenotypes may vary by race and ethnicity, so does the effectiveness of therapies for CVD and associated risk factors.

Clinicians have long recognized that antihypertensives have disparate blood pressure lowering effects by race/ethnicity [25], yet few observational studies or clinical trials have been designed to directly compare effectiveness across these strata. In fact, application of therapies without a trial has led to inappropriate treatment guidelines. For example, the South Asian ethnicity is characterized by high triglycerides, low HDL cholesterol, high propensity to diabetes, and low physical activity [26]. Based on prior Western guidelines, the Indian Society of Hypertension advocated beta-blockers (which have all the side effects mentioned above) as the first line treatment [27]. Furthermore, nuanced effects of combination antihypertensive therapy are often not apparent without race- or ethnicity-specific data; secondary subgroup analyses have demonstrated preliminary evidence for variable blood pressure-lowering results upon addition of a second antihypertensive agent, depending not only on the pharmacologic regimen but also on race/ethnicity [28]. The recently completed CREOLE study represents one of the only examples of a study whose primary aim was to determine race-specific treatment strategies for high blood pressure. This study found that combination regimens, including calcium channel blockers, were more effective than a regimen based on renin-angiotensin system blockade and thiazide diuretics in sub-Saharan Africa [29]. None of these studies have been launched to compare initial blood pressure treatment strategies in other races or ethnicities. Clearly, the differential effects of pharmacologic agents on blood pressure by race can influence the downstream risk for CVD events, perhaps most famously shown by the Antihypertensive and Lipid-Lowering Treatment to Prevent Heart Attack Trial (ALLHAT), in which African Americans in the renin-angiotensin blockers arm experienced higher risk for strokes and other CVD events [30].

With the emergence of evidence that South Asians exhibit a different diabetes phenotype [19], the choice of pharmacologic therapy for glycemic control in diabetes or pre-diabetes should ideally account for ethnic factors. Data suggest that dipeptidyl peptidase-4 inhibitors are more effective at lowering hemoglobin A1c values and fasting plasma glucose in Asians versus non-Asians with type 2 diabetes mellitus [31]. Differences in glycemic response exist even in patients with impaired glucose tolerance; in a study of patients treated with rosiglitazone for prevention of diabetes, the magnitude of risk reduction for developing diabetes was mediated by ethnicity, with Hispanics experiencing a much greater risk reduction than South Asians [32]. While rosiglitazone is no longer widely used, the ethnicity and effectiveness interaction observed in the DREAM trial supports that antidiabetic medications in other classes should undergo similar evaluations. Although the effectiveness of sodium glucose co-transporter 2 inhibitors has not been shown to significantly differ by race [33, 34], a larger number of well-powered studies of diverse populations are needed to validate this finding.

Perhaps as important as the differential effects of pharmacologic therapies for CVD are the sociocultural context and healthcare systems in which these therapies are delivered. Reliable access to healthcare in LMICs, particularly in rural areas, continues to be a challenge and, in contrast to high-income countries, the average person has minimal interaction with primary care providers who could conduct CVD risk screening. However, efforts are ongoing to improve primary healthcare delivery in LMICs, with community health outreach models that task-shift to local health workers showing success [35]. Recent meta-analyses have found substantial evidence for the effectiveness of lifestyle interventions, key examples of which have led to a twofold higher likelihood of tobacco cessation [36] or improved knowledge regarding complications of type 2 diabetes [37]. These innovative lower-cost models have high potential, particularly to engage with evolving technologies such as artificial intelligence, and may inform the evolving role of ‘physician-extenders’ in high-income countries. However, they nonetheless require integration within a larger, better supported healthcare system [38]. Many LMICs are making ambitious steps towards universal health care [39, 40], with Thailand integrating country-specific, health technology cost-effectiveness assessments into the development of a successfully implemented universal healthcare package [41].

Devoting more resources to the healthcare system is not enough on its own to improve CVD care; rather, awareness and integration of cultural norms and family structures into treatment plans is also necessary to provide effective care, especially that targeted at prevention and/or lifestyle modification. Interventions for physical activity in Brazil, for example, may be more effective if inclusive of football/soccer, since recreational football/soccer use is common in men and holds a positive cultural value [42], whereas other forms (e.g., yoga) may be more acceptable for South Asians [43]. Family members and other social support networks are crucial mediators of dietary and physical activity interventions as well as of medication adherence, especially for type 2 diabetes [44]. Clearly, these ecosystems and their interplay with the patient vary across the world, with different family members, for example, making food-purchasing or leisure-time activity decisions. Not only would the intervention design need to be tailored, but the relative effectiveness may vary depending on the region and/or race/ethnicity. Thus, a specific region may find it more cost-effective and worthwhile to invest in family-based lifestyle interventions, whereas another may want to focus on medication availability and delivery.

## Conclusion

Since CVDs are recognized as a major cause of poor health in all regions of the world, researchers now have a strong rationale to investigate the differences in risk factors and management strategies. Data increasingly support the likelihood that genetic, cultural, and environmental variations exist in causes of CVD by race and ethnicity. These variations should be accounted for when devising strategies for the prevention and treatment of CVD. Clinical trials and observational studies designed specifically to probe differences across race/ethnicity strata are crucial to improving CVD care across the world.

## Data Availability

Not applicable.

## References

[1] Stamler J, Neaton JD (2008). The multiple risk factor intervention trial (MRFIT) – importance then and now. JAMA..

[2] Kannel WB, Wolf PA (2008). Framingham study insights on the hazards of elevated blood pressure. JAMA..

[3] King P, Peacock I, Donnelly R (1999). The UK prospective diabetes study (UKPDS): clinical and therapeutic implications for type 2 diabetes. Br J Clin Pharmacol.

[4] Hofman A, Grobbee DE, de Jong PT, van den Ouweland FA (1991). Determinants of disease and disability in the elderly: the Rotterdam elderly study. Eur J Epidemiol.

[5] Prabhakaran D, Anand S, Watkins D (2018). Cardiovascular, respiratory, and related disorders: key messages from disease control priorities, 3rd edition. Lancet..

[6] Patel V, Chisholm D, Dua T, Laxminarayan R, Medina-Mora ME, editors. 2015. Mental, Neurological, and Substance Use Disorders. Disease Control Priorities, third edition, volume 4. Washington, DC: World Bank. License: Creative Commons Attribution CC BY 3.0 IGO. 10.1596/978-1-4648-0426-7. 27227198

[7] Klag MJ, Whelton PK, Randall BL, Neaton JD, Brancati FL, Stamler J (1997). End-stage renal disease in African-American and white men. 16-year MRFIT findings. JAMA..

[8] Fogo A, Breyer JA, Smith MC (1997). Accuracy of the diagnosis of hypertensive nephrosclerosis in African Americans: a report from the African American study of kidney disease (AASK) trial. AASK Pilot Study Investigators. Kidney Int.

[9] Sorof JM, Hawkins EP, Brewer ED, Boydstun II, Kale AS, Powell DR (1998). Age and ethnicity affect the risk and outcome of focal segmental glomerulosclerosis. Pediatr Nephrol.

[10] Genovese G, Friedman DJ, Ross MD (2010). Association of trypanolytic ApoL1 variants with kidney disease in African Americans. Science..

[11] Tzur S, Rosset S, Shemer R (2010). Missense mutations in the APOL1 gene are highly associated with end stage kidney disease risk previously attributed to the MYH9 gene. Hum Genet.

[12] Thomson R, Genovese G, Canon C (2014). Evolution of the primate trypanolytic factor APOL1. Proc Natl Acad Sci U S A.

[13] Freedman BI, Moxey-Mims M (2018). The APOL1 long-term kidney transplantation outcomes network – APOLLO. Clin J Am Soc Nephrol.

[14] Anand S, Zheng Y, Montez-Rath ME (2017). Do attributes of persons with chronic kidney disease differ in low-income and middle-income countries compared with high-income countries? Evidence from population-based data in six countries. BMJ Glob Health.

[15] Suzuki H, Kiryluk K, Novak J (2011). The pathophysiology of IgA nephropathy. J Am Soc Nephrol.

[16] Mercader JM, Florez JC (2017). The genetic basis of type 2 diabetes in Hispanics and Latin Americans: challenges and opportunities. Front Public Health.

[17] Consortium STD, Williams AL, Jacobs SB (2014). Sequence variants in SLC16A11 are a common risk factor for type 2 diabetes in Mexico. Nature..

[18] Nair AK, Baier LJ (2015). Complex genetics of type 2 diabetes and effect size: what have we learned from isolated populations?. Rev Diabet Stud.

[19] Staimez LR, Deepa M, Ali MK, Mohan V, Hanson RL, Narayan KMV (2019). Tale of two Indians: heterogeneity in type 2 diabetes pathophysiology. Diabetes Metab Res Rev.

[20] Vartiainen E, Du DJ, Marks JS (1991). Mortality, cardiovascular risk factors, and diet in China, Finland, and the United States. Public Health Rep.

[21] Ueshima H, Zhang XH, Choudhury SR (2000). Epidemiology of hypertension in China and Japan. J Hum Hypertens.

[22] Corsi DJ, Subramanian SV, Chow CK (2013). Prospective Urban Rural Epidemiology (PURE) study: baseline characteristics of the household sample and comparative analyses with national data in 17 countries. Am Heart J..

[23] Yusuf S, Rangarajan S, Teo K (2014). Cardiovascular risk and events in 17 low-, middle-, and high-income countries. N Engl J Med.

[24] Caplin B, Yang CW, Anand S (2019). The International Society of Nephrology's international Consortium of collaborators on chronic kidney disease of unknown etiology: report of the working group on approaches to population-level detection strategies and recommendations for a minimum dataset. Kidney Int.

[25] James PA, Oparil S, Carter BL (2014). 2014 evidence-based guideline for the management of high blood pressure in adults: report from the panel members appointed to the eighth joint National Committee (JNC 8). JAMA..

[26] Karthikeyan G, Teo KK, Islam S (2009). Lipid profile, plasma apolipoproteins, and risk of a first myocardial infarction among Asians: an analysis from the INTERHEART study. J Am Coll Cardiol.

[27] Association of Physicians of India (2013). Indian guidelines on hypertension (I.G.H.) - III. 2013. J Assoc Physicians India.

[28] Gupta AK, Poulter NR, Dobson J (2010). Ethnic differences in blood pressure response to first and second-line antihypertensive therapies in patients randomized in the ASCOT trial. Am J Hypertens.

[29] Ojji DB, Mayosi B, Francis V (2019). Comparison of dual therapies for lowering blood pressure in black Africans. N Engl J Med.

[30] ALLHAT Officers and Coordinators for the ALLHAT Collaborative Research Group (2002). The Antihypertensive and Lipid-Lowering Treatment to Prevent Heart Attack Trial. Major outcomes in high-risk hypertensive patients randomized to angiotensin-converting enzyme inhibitor or calcium channel blocker vs diuretic: the antihypertensive and lipid-lowering treatment to prevent heart attack trial (ALLHAT). JAMA..

[31] Kim YG, Hahn S, Oh TJ, Kwak SH, Park KS, Cho YM (2013). Differences in the glucose-lowering efficacy of dipeptidyl peptidase-4 inhibitors between Asians and non-Asians: a systematic review and meta-analysis. Diabetologia..

[32] Boyko EJ, Gerstein HC, Mohan V (2010). Effects of ethnicity on diabetes incidence and prevention: results of the diabetes REduction assessment with ramipril and rosiglitazone medication (DREAM) trial. Diabet Med.

[33] Neal B, Perkovic V, Mahaffey KW (2017). Canagliflozin and cardiovascular and renal events in type 2 diabetes. N Engl J Med.

[34] Zinman B, Wanner C, Lachin JM (2015). Empagliflozin, cardiovascular outcomes, and mortality in type 2 diabetes. N Engl J Med.

[35] Prabhakaran D, Ajay VS, Tandon N (2019). Strategic opportunities for leveraging low-cost, high-impact technological innovations to promote cardiovascular health in India. Ethn Dis.

[36] Jeet G, Thakur JS, Prinja S, Singh M (2017). Community health workers for non-communicable diseases prevention and control in developing countries: evidence and implications. PLoS One.

[37] Alaofe H, Asaolu I, Ehiri J (2017). Community health workers in diabetes prevention and management in developing countries. Ann Glob Health.

[38] Schneider H, Okello D, Lehmann U (2016). The global pendulum swing towards community health workers in low- and middle-income countries: a scoping review of trends, geographical distribution and programmatic orientations, 2005 to 2014. Hum Resour Health.

[39] Prabhakaran D, Jaacks LM (2019). Reflections from India on scaling up risk factor control for cardiovascular diseases to reach 1 billion adults. Circulation..

[40] Jamison DT, Alwan A, Mock CN (2018). Universal health coverage and intersectoral action for health: key messages from disease control priorities, 3rd edition. Lancet..

[41] Tangcharoensathien V, Witthayapipopsakul W, Panichkriangkrai W, Patcharanarumol W, Mills A (2018). Health systems development in Thailand: a solid platform for successful implementation of universal health coverage. Lancet..

[42] Lima DF, Piovani VGS, Lima LA (2018). Recreational soccer practice among adults, in Brazilian capitals, 2011–2015. Epidemiol Serv Saude.

[43] Innes KE, Bourguignon C, Taylor AG (2005). Risk indices associated with the insulin resistance syndrome, cardiovascular disease, and possible protection with yoga: a systematic review. J Am Board Fam Pract.

[44] Young-Hyman D, de Groot M, Hill-Briggs F, Gonzalez JS, Hood K, Peyrot M (2016). Psychosocial care for people with diabetes: a position statement of the American Diabetes Association. Diabetes Care.

